# Diaphragmatic pheochromocytoma: Two case reports and a review of the literature

**DOI:** 10.1097/MD.0000000000040939

**Published:** 2024-12-13

**Authors:** Xinsheng Xi, Guanghui Yan, Baihong Guo, Gang Jin, Chenming Guo, Bin Feng

**Affiliations:** a Department of Urology, Gansu Provincial Hospital, Lanzhou, China; b Department of Ophthalmology, The First Hospital of Lanzhou University, Lanzhou, China; c Department of Thoracic Surgery, Gansu Provincial Hospital, Lanzhou, China.

**Keywords:** ectopic pheochromocytomas/paragangliomas, endoscopic surgery, transperitoneal, transthoracic

## Abstract

**Rationale:**

Pheochromocytomas typically arise in the adrenal medulla, whereas ectopic pheochromocytomas/paragangliomas commonly occur near the abdominal aorta, bladder, mediastinum, and head. Diaphragmatic pheochromocytomas are exceedingly rare, and there is limited surgical experience with their treatment.

**Patient concerns:**

In Case A, the subject is a 45-year-old male, while in Case B, the subject is a 59-year-old female. Both patients present with a history of paroxysmal hypertension. Computed tomography imaging revealed the presence of diaphragmatic tumors in both patients. The tumor in Case A was observed in the left diaphragm, while the tumor in Case B was located in the right diaphragm.

**Diagnoses:**

Two patients were diagnosed with diaphragmatic pheochromocytoma on the basis of disease progression, imaging, endocrinological assessment and postoperative histopathological examination.

**Intervention:**

Both patients received the same standardized preoperative preparation, which included hypotensive therapy and intravenous rehydration. Subsequently, Case A underwent a transabdominal robotic laparoscopic surgical resection, while Case B underwent a transthoracic thoracoscopic surgical resection.

**Outcomes:**

Both patients demonstrated a favorable recovery trajectory and exhibited stable blood pressure at the 3-month follow-up.

**Lessons:**

This report serves to remind the reader that the transthoracic approach to diaphragmatic pheochromocytoma may prove to be more advantageous than the transperitoneal approach. Additionally, precise preoperative localization of the tumor and careful intraoperative monitoring and assessment are imperative to achieve favorable outcomes.

## 1. Introduction

Pheochromocytomas only occur in 0.1% of hypertensive patients. These tumors typically arise in the adrenal medulla, but approximately 25% of cases involve ectopic pheochromocytomas/paragangliomas located outside the adrenal glands.^[1]^ The most common locations for ectopic pheochromocytomas/paragangliomas include the vicinity of the abdominal aorta, bladder, mediastinum, and head.^[2]^ Due to the consistent pathogenic mechanism involving the secretion and release of excessive catecholamine hormones, the clinical presentation closely resembles that of adrenal pheochromocytoma, manifesting in a variety of symptoms, such as paroxysmal hypertension, palpitations, headache, and sweating. Imaging modalities, such as computed tomography (CT) or magnetic resonance imaging, are commonly used for tumor localization, while biochemical diagnosis plays a crucial role in identification. The latter is obtained through assessment of 24-hour urine vanillylmandelic acid (VMA) and plasma epinephrine.^[3]^ Surgery is recommended as the primary treatment for locoregional diseases.^[4]^ This article presents the treatment of 2 uncommon cases of diaphragmatic pheochromocytoma and evaluates 2 surgical approaches.

## 2. Case presentation

### 2.1. Case A

**Figure 1.**
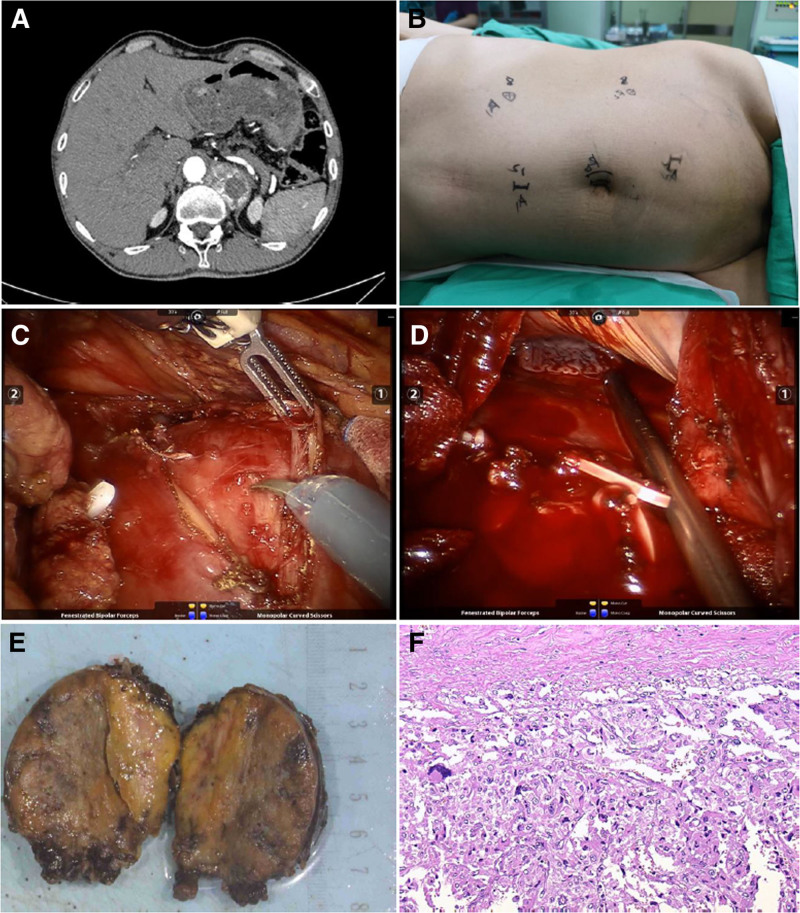
Radiological, surgical procedures, and pathologic diagnosis in Case A. (A) The CT showed an ovoid mass in the plane of the area between T11 and T12 vertebrae, within the left septum of the spine. (B) The observation mirror was positioned on the left side next to the umbilicus, operating arm No. 2 was placed 2 cm below the rib margin in the midclavicular line, operating arm No. 1 was placed 10 cm above the anterior superior iliac spine, the assistant’s channel was placed 5 cm away from the umbilicus to the lateral side of the pubic symphysis, and the alternate assistant’s channel was placed 10 cm above the umbilicus. (C) Incision of the diaphragm to expose the tumor. (D) Diaphragm rupture. (E) Surgical specimen of the ectopic pheochromocytoma. (F) Pathological findings of the resected specimen: Haematoxylin and eosin (HE) staining lower-power field. CT = computed tomography, HE = haematoxylin and eosin.

A 45-year-old male patient presented with palpitations, headache, vertigo, and paroxysmal hypertension for 5 months. Upon examination, his blood pressure was 200/145 mm Hg, and heart rate ranged from 130 to 90 beats per minute. Imaging studies revealed a 65 × 56 × 45 mm ovoid mass located in the left septum of the spine between the T11 and T12 vertebrae on upper abdominal CT, suggestive of a neurogenic tumor with no evidence of metastasis (Fig. 1A). Laboratory findings revealed urine VMA levels of 17.28 mg/24 hour, standing renin levels of 242.63 ng/L, and recumbent renin levels of 131.48 ng/L. No abnormalities were observed in standing or recumbent aldosterone, standing or recumbent angiotensin, plasma cortisol, pro-adrenocorticotropic hormone, plasma creatinine, and urea nitrogen levels. Prior to the surgical procedure, the patient was administered oral phenoxybenzamine, starting at an initial dose of 10 mg/day and gradually increasing to 40 mg/day, which was determined to be the optimal dosage. Following 4 weeks of maintenance of this dosage, the patient’s blood pressure stabilized at approximately 140/80 mm Hg. Metoprolol at a dosage of 25 mg/day was used to maintain a heart rate below 90 beats per minute. Intravenous fluid replacement was administered 1 week prior to the surgical procedure to prevent postoperative hypotension.

The da Vinci Si Robotic System was used for the purpose of conducting tumor resection through the peritoneal cavity. Following intravenous anesthesia, the patient was placed in the right lateral decubitus position with elevation of the waist. Trocars were inserted as shown in Figure 1B. Notably, no tumor was visualized in the left adrenal region during surgical intervention.

Our findings indicated localized elevation of the left diaphragm without any surface abnormalities. Incision of the diaphragm along the elevated diaphragm revealed a vascularized ovoid tumor (Fig. 1C). The patient experienced significant fluctuations in blood pressure during tumor isolation, which was successfully removed by careful separation of the fibrous tissue and ligating vessels. However, a 3 cm rupture of the diaphragm was observed postoperatively (Fig. 1D). The diaphragm was sutured and the patient’s lungs were inflated to expel gas in the chest prior to closure of the chest cavity. An abdominal drain was inserted to complete the procedure, and the patient was subsequently transferred to the ward. Postoperative pathology revealed that the lesion primarily consisted of chromaffin cells (Fig. 1E, F).

### 2.2. Case B

**Figure 2.**
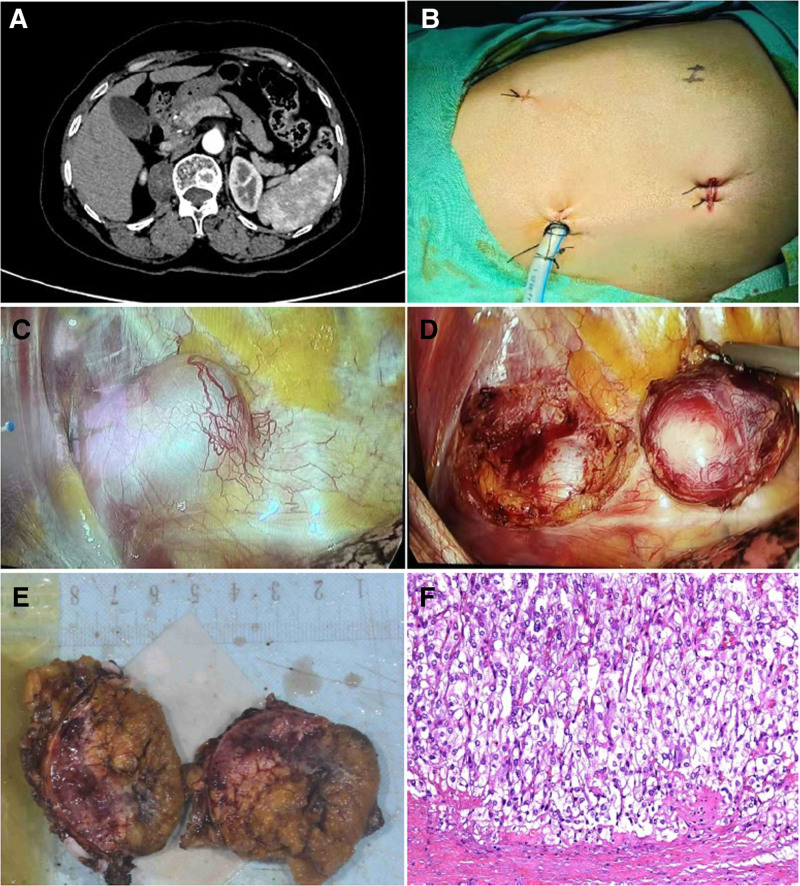
Radiological, surgical procedures, and pathologic diagnosis in Case B. (A) The CT showed a roundish slightly hypodense lesion adjacent to the spine in the right diaphragmatic angle. (B) The main surgical incision is 4th intercostal space along the midaxillary line; the obersvation hole is 7th intercostal space along the midaxillary line, and the auxiliary incision is 9th intercostal space along the subscapular line. (C) Exposure of the tumor. (D) Remove the tumor. (E) Surgical specimen of the ectopic pheochromocytoma. (F) Pathological findings of the resected specimen: Haematoxylin and eosin (HE) staining lower-power field. CT = computed tomography, HE = haematoxylin and eosin.

A 59-year-old female, had presented with back pain, and a mass was identified in the right diaphragm during an examination 1 week prior to examination. She had a history of hypertension for the past 5 years and was being treated with nifedipine. Despite having a blood pressure and heart rate within normal limits, the patient exhibited occasional palpitations, headaches, vertigo, and paroxysmal hypertension. Abdominal CT revealed a 55 × 41 × 37 mm roundish slightly hypodense lesion near the spine in the right diaphragmatic angle, with mild persistent enhancement observed in the center of the lesion on the enhancement scan diagram (Fig. 2A). Laboratory results revealed a urine VMA level of 12.38 mg/24 hour, a standing renin level of 5.38 ng/L, and a recumbent renin level of 2.16 ng/L. No abnormalities were observed in standing or recumbent aldosterone, standing or recumbent angiotensin, plasma cortisol, pro-adrenocorticotropic hormone, plasma creatinine, and urea nitrogen levels. Preoperative preparations were performed as described previously.

In contrast to previous cases, thoracic surgeons were invited to perform the thoracoscopic procedures. Following anesthesia, the patient was placed in the left lateral decubitus position. Thoracoscopic port placements are depicted in Figure 2B, revealing an ovate mass located at the posterior inferior costophrenic angle within the right thoracic cavity (Fig. 2C). The tumor was skillfully excised by meticulous resection along its base, subsequent to incision of the mediastinal pleura adjacent to the tumor (Fig. 2D). Suturing was performed to repair the injured pleura, and the thoracic cavity was assessed for any air leak. A drainage tube was inserted through the trocar channel and linked to a closed thoracic drainage device. After completing layer-by-layer suturing of the incision, the patient was transferred to the ward.

Subsequent pathological analysis confirmed the diagnosis of paraganglioma. (Fig. 2E, F).

The study was approved by the Ethics Committee of Gansu Provincial Hospital. The article and author have obtained written informed consent from 2 patients for the publication of their medical records, case details and images.

## 3. Discussion

The presence of ectopic pheochromocytomas/paragangliomas is strongly suspected when tumors are located in the retroperitoneum in close proximity to the abdominal paraganglia, spine, or near the renal hilum, even in the absence of typical symptoms, such as headache, palpitations, or hypertension.^[5]^ Surgical resection of catecholamine-secreting tumors is a complex and high-risk procedure that requires the expertise of a skilled surgical team and anesthesiologist. Prior to the surgical procedure, it is imperative for surgeons to assess the adequacy of adrenergic blockade. Effective communication between surgeons and anesthesiologists is essential during surgery, particularly during tumor dissection and resection, to closely monitor fluctuations in blood pressure, heart rate, and hemodynamics. Minimally invasive surgery is generally favored; however, open surgery may be necessary for pheochromocytomas > 6 cm with concomitant adherent tumor that are difficult to separate, or in cases where the surgeon lacks proficiency; alternatively, a strategy of diagnostic laparoscopy followed by conversion to open procedure or hand-assisted resection may be employed if necessary.^[6]^ When a pheochromocytoma disrupts the operative environment of other tumors, it is imperative to prioritize resection to achieve hemodynamic stability. Additionally, the simultaneous resection of concurrent tumors often results in a heightened incidence of complications. Therefore, a 2-stage resection approach may be more suitable for the management of multiple tumors.^[7]^

Diaphragmatic pheochromocytomas are exceedingly rare, as ectopic pheochromocytomas are typically found within the abdominal cavity.^[8]^ To the best of our knowledge, there have been only 3 previous clinical case reports on this topic.^[9–11]^ Wu et al^[10]^ reported a case of diaphragmatic pheochromocytoma resection using the transabdominal approach, while the other 2 clinical cases did not specify the surgical approach. In transabdominal laparoscopic surgery, the tumor may be obscured by the diaphragm, rendering it invisible in the surgical field, and potentially overlooked. Instances have been reported in patients with diaphragmatic pheochromocytomas in whom the primary tumor was not identified during the initial surgery.^[9]^

The same dilemma encountered during surgery in the first case was the absence of a mass within the upper pole of the kidney to the diaphragm. However, a breakthrough was achieved when an incision through the diaphragm unveiled the concealed tumor. In light of the challenges encountered during the Case A procedure, including abdominal organ interference and difficulty in exposing the surgical area, the Case B procedure was performed via a thoracoscopic transthoracic approach for tumor resection, in accordance with the recommendations of the thoracic surgeon. It was discovered that tumor visualization was more straightforward during surgery via the transthoracic route than anticipated, due to the atrophy of the lung tissue. A comparative analysis of treatment outcomes for both cases is presented in Table 1. It is important to note that the interpretation of these findings was constrained by the limited number of cases. Nevertheless, preliminary evidence suggests that the transthoracic approach may have advantages over the transabdominal approach. It is anticipated that additional similar cases will be documented in the future, thereby furnishing ample clinical data.

**Table 1:** Comparison of 2 cases.

	Case A	Case B
Gender	Male	Female
Age (yr)	45	59
Weight (kg)	64	66
BMI	21.4	25.8
Sides	Left	Right
Tumor size (mm)	65 × 56 × 45	55 × 41 × 37
Approach	Transperitoneal	Transthoracic
Surgical equipment	Robotic laparoscopy	Thoracoscopy
Duration of surgery (h)	5	1
Intraoperative paroxysmal hypertension	Yes	No
Bleeding volume (mL)	350	10
Diaphragmatic perforation	Yes	No
Intrathoracic drain	No	Yes
Postoperative blood pressure	Normal	Abnormal

BMI = body mass index.

Conventional wisdom suggests that the lack of haptic feedback in robotic surgical systems may result in inadvertent pressure being applied to the tumor, which could potentially lead to a rapid release of catecholamines into the bloodstream and subsequent fluctuations in blood pressure. Nevertheless, the utilization of robotic access has been documented to confer advantages in the context of pheochromocytoma surgery.^[12]^ Our experience has demonstrated that robotic surgery is a safer method for the removal of pheochromocytoma than standard laparoscopic surgery. This can be attributed to the extremely high stability of the robotic arm.

The postoperative outcomes of patients with pheochromocytomas/paragangliomas are influenced by preoperative measures, including reduction of blood pressure, heart rate, and expansion of blood volume.^[13]^ Patients who undergo adequate preoperative preparation have a mortality rate ranging from 0% to 3%, whereas those who do not undergo such preparation may have a mortality rate as high as 43%.^[14]^ Based on the most recent classification by the World Health Organization, all pheochromocytomas/paragangliomas have the potential to metastasize, although the specific term “malignant” is no longer explicitly used.^[15]^ A study of patients with pheochromocytoma revealed a recurrence rate of approximately 20% among those with ectopic pheochromocytomas 5 years postsurgery.^[16]^ It is therefore imperative that a comprehensive and long-term follow-up programme is implemented for each patient.

## 4. Conclusion

There is a paucity of research on targeted treatment strategies for ectopic pheochromocytomas/paragangliomas, and a standardized surgical protocol for diaphragmatic pheochromocytomas is currently lacking. In cases of this particular tumor, surgical intervention utilizing the transthoracic approach in collaboration with a thoracic surgeon may be preferable course of action. Furthermore, accurate preoperative tumor localization and meticulous intraoperative monitoring are imperative to achieve favorable surgical outcomes.

## Acknowledgments

This study was financially supported by the Lanzhou Science and Technology Bureau (Project number: 2022-3-2). The authors declare that there is no conflict of interest.

## Author contributions

**Project administration:** Baihong Guo, Gang Jin, Chenming Guo.

**Writing – review & editing:** Xinsheng Xi, Bin Feng.

**Writing – original draft:** Guanghui Yan.
